# Blackcurrant Supplementation Improves Trabecular Bone Mass in Young but Not Aged Mice

**DOI:** 10.3390/nu10111671

**Published:** 2018-11-05

**Authors:** Junichi Sakaki, Melissa Melough, Sang Gil Lee, Judy Kalinowski, Sung I. Koo, Sun-Kyeong Lee, Ock K. Chun

**Affiliations:** 1 Department of Nutritional Sciences, University of Connecticut, Storrs, CT 06269, USA; junichi.sakaki@uconn.edu (J.S.); Melissa.melough@uconn.edu (M.M.); sung.koo@uconn.edu (S.I.K.); 2 Department of Food Science and Nutrition, Pukyong National University, Busan 48513, Korea; sglee1125@pknu.ac.kr; 3 Center on Aging, University of Connecticut Health Center, Farmington, CT 06030, USA; kalinowski@uconn.edu

**Keywords:** blackcurrants, flavonoids, anthocyanins, antioxidant, oxidative stress, inflammation, bone health, osteoblasts, osteoclasts

## Abstract

Due to deleterious side effects of currently available medications, the search for novel, safe, and effective preventive agents for improving bone health in aging continues and is urgently needed. This study aimed to determine whether dietary blackcurrants (BC), an anthocyanin-rich berry, can improve bone mass in a mouse model of age-related bone loss. Thirty-five female C57BL/6J mice, 3 months old (*n* = 20) and 18 months old (*n* = 15), were randomized to consume either a standard chow diet or a standard chow diet with 1% (*w*/*w*) BC for four months. Dual-energy X-ray absorptiometry, Micro computed tomography (µCT), and histomorphometric analyses were conducted to assess bone parameters on femurs. Biochemical assays were conducted to determine bone resorption, antioxidant activity, and inflammation in humerus homogenates. Trabecular bone volume (BV/TV) was significantly lower in aged mice compared to young mice (young control, 3.7 ± 0.4% vs aged control, 1.5 ± 0.5%, mean ± SEM (standard error of mean), *p* < 0.01; young BC, 5.3 ± 0.6% vs aged BC, 1.1 ± 0.3%, *p* < 0.001). µCT analysis revealed that BC supplementation increased trabecular BV/TV in young mice by 43.2% (*p* < 0.05) compared to controls. Histomorphometric analysis revealed a 50% increase, though this effect was not statistically significant (*p* = 0.07). The osteoblast surface increased by 82.5% in aged mice with BC compared to controls (*p* < 0.01). In humerus homogenates of young mice, BC consumption reduced C-telopeptide of type I collagen by 12.4% (*p* < 0.05) and increased glutathione peroxidase by 96.4% (*p* < 0.05). In humerus homogenates of aged mice, BC consumption increased catalase by 12% (*p* = 0.09). Aged mice had significantly elevated concentrations of tumor necrosis factor α (TNF-α), a pro-inflammatory cytokine contributing to bone resorption, which was reduced by 43.3% with BC consumption (*p* = 0.06). These results suggest that early consumption of BC may protect from aging-associated bone loss.

## 1. Introduction

Age-related bone loss is characterized by a decline in bone mass and weakening of bone microarchitecture that accelerates with aging [1]. In advanced age, the rate of bone resorbed by the basic multicellular unit (BMU), composed of bone-resorbing osteoclasts and bone-forming osteoblasts, outpaces the rate of bone formed, resulting in a negative BMU imbalance [2,3,4]. Compared to cortical bone, trabecular bone is rapidly lost due to the thin plates of mineralized matrix being easily perforated, as well as the large surface area of the trabeculae that facilitates remodeling [5,6]. This causes similar absolute losses of cortical and trabecular bone during the first ten years of menopause, despite cortical bone composing 80% of the skeleton and trabecular bone 20% [7]. Several factors associated with aging, such as increased oxidative stress, inflammation, and sex hormone deficiency, exacerbate bone loss [8,9,10]. Common treatment strategies for improving bone mass and reducing fracture risk include directly targeting bone resorption or bone formation with anti-resorptive and bone-anabolic medications, respectively, as well as prophylactic procedures such as fall prevention. The pharmacologic agents, while effective at improving bone mass, have potential adverse effects such as upper gastrointestinal symptoms, hypocalcemia, atypical femoral fractures, osteonecrosis of the jaw, increased risk of coronary heart disease, stroke and dementia; additionally, contraindications exist for each type of medication [11,12,13]. In light of these risks and limitations to pharmaceutical treatment, nutritional therapies are being investigated as safe alternatives or complements to bone restoration therapy. Treatments such as calcium and vitamin D supplementation have the potential to directly impact bone metabolism, and other nutritional interventions may also modulate contributors to age-related bone loss such as oxidative stress and inflammation [14].

Recently, foods rich in flavonoids, a class of secondary plant metabolites abundant in certain fruits and vegetables, have been investigated for their potential to improve bone mass. Our research group has previously shown that blackcurrants (BC) (*Ribes nigrum*) possess the highest concentration of anthocyanins, a subclass of flavonoids, amongst commonly consumed berries such as blackberries and blueberries [15]. The anthocyanin content of BC is composed of delphinidin (74% of total anthocyanins) and cyanidin (26%) [15], and delphinidin has been found to prevent bone resorption [16]. Animal studies [17,18,19] have demonstrated that certain fruits rich in anthocyanins improve bone mass. However, these studies utilized an ovariectomy (OVX)-induced osteoporosis model, which mimics postmenopausal estrogen deficiency but does not specifically mimic the effects of aging. Thus, it is difficult to apply the findings from OVX-induced osteoporosis to age-related bone loss. In this study, we utilized mice that are advanced in age but otherwise healthy in order to more accurately model the effects of aging. Given the potential adverse effects of pharmacological agents in the treatment of age-related bone loss, we sought to evaluate a natural and safer dietary approach. This study aimed to determine whether dietary BC, an anthocyanin-rich berry, can improve bone mass in a mouse model of age-related bone loss. We hypothesized that BC consumption would improve bone mass in both young and aged mice compared to the control groups.

## 2. Methods

### 2.1. Animals and Diets

**Figure 1:**
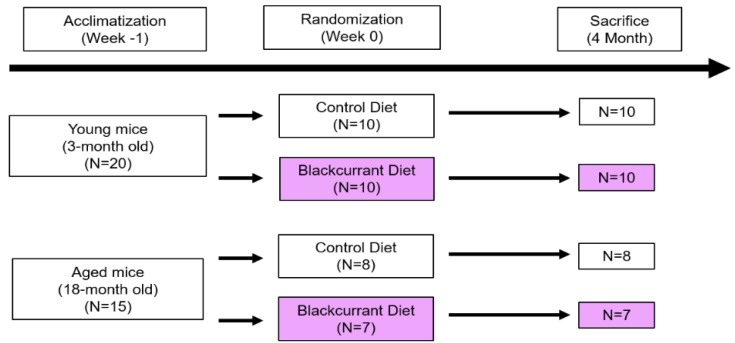
Experimental design.

Forty female C57BL/6J mice, 3 months old (*n* = 20) and 18 months old (*n* = 20), obtained from the National Institute on Aging (Bethesda, MD), were housed five per cage in a temperature- and humidity-controlled room in the Center for Laboratory Animal Care at the University of Connecticut. Female mice were selected as the animal model in order to draw comparisons with previous studies [16,17,20,21,22,23,24]. After a one-week acclimatization period, mice were randomized to consume either a standard chow diet (control) or a standard chow diet with 1% (*w*/*w*) anthocyanin-rich BC extract (experimental), provided by Just the Berries Ltd. (Palmerston North, New Zealand), *ad libitum*, resulting in four treatment groups (young control, young BC, aged control, aged BC) as shown in Figure 1. Four of the 18-month-old mice died throughout the study and one developed ulcerative dermatitis and was consequently excluded from the analysis, leaving fifteen 18-month-old mice for the final analysis. All mice consumed their assigned diets for four months. Body weight and food intake were measured weekly. All protocols related to animal care and handling were approved by the Institutional Animal Care and Use Committee from the University of Connecticut and the University of Connecticut Health Center (protocol #100829-0117, approved 11/16/2015).

After four months, mice were fasted overnight and sacrificed by CO_2_ and cervical dislocation. Right femurs were removed and fixed in 70% ethanol at 4 °C for microcomputed tomography (µCT) and left femurs in 4% paraformaldehyde in phosphate buffered saline (PBS) for histomorphometric analyses. Humeri were collected and stored at −80 °C for biomarker assays.

### 2.2. Bone Microcomputed Tomography (µCT) Analysis 

The right femur of each mouse was isolated and fixed in 70% ethanol and analyzed by high-resolution µCT (µCT 40; Scanco Medical, AG, Basserdorf, Switzerland). Trabecular morphometry was assessed for bone volume fraction (BV/TV), trabecular thickness (Tb.Th), trabecular number (Tb.N), and trabecular spacing (Tb.Sp). Cortical morphometry was assessed for the segmented bone area. Trabecular and cortical morphometry were quantified within the metaphyseal region of the distal femur, and representative three-dimensional images were constructed. The measurement terminology and units used for μCT analysis were those recommended by Bouxsein et al. [25].

### 2.3. Bone Histomorphometric Analysis

The left femur of each mouse was fixed in 4% paraformaldehyde (Sigma-Aldrich, St. Louis, MO, USA), decalcified, embedded in paraffin, and sectioned serially at a 7-µm thickness, then stained with hematoxylin and eosin or tartrate-resistant acid phosphatase. Endpoints were trabecular and cellular bone parameters. Osteoclast and osteoblast parameters were expressed as a percentage of total bone surface: osteoclast surface/bone surface (Oc.S/BS), osteoblast surface/bone surface (Ob.S/BS), and eroded surface/bone surface (ES/BS). Histomorphometric analyses were quantified 200 μm underneath growth plates and cortical bones based on recommendations by the Nomenclature Committee of the American Society for Bone and Mineral Research [26] using Osteomeasure (Nashville, TN, USA).

### 2.4. Dual-Energy X-Ray Absorptiometry (DXA) Analysis

DXA was used to measure bone mineral density (BMD) and content (BMC) of the femur in young and aged mice that consumed either the control or BC diet (PIXImus bone densitometer with dedicated software for analysis of small animals; Lunar Corp., Madison, WI, USA). The femurs from each animal were removed and preserved in 70% ethanol until DXA measurement.

### 2.5. Bone Homogenate Biomarkers 

The humerus of each mouse was prepared by first gently removing adhering tissue with a scalpel. The humerus was then placed in 500 µL of PBS and homogenized for fifteen seconds. The homogenate was centrifuged at 14,000× *g* at 4 °C for ten minutes and the supernatant was collected and stored at −80 °C until used for biomarker assays.

Commercial kits were used according to manufacturers’ instructions to measure bone homogenate biomarkers. C-terminal telopeptide of type I collagen (CTX) was measured with an enzyme-linked immunosorbent assay (ELISA) kit (MBS453660, MyBioSource, Inc., San Diego, CA, USA) to determine bone resorption. Glutathione peroxidase (GPx) (#703102, Cayman Chemical, Ann Arbor, MI, USA), and catalase (CAT) (Cayman, #707002) were measured to determine the antioxidant capacity. Tumor necrosis factor α (TNF-α) (R&D Systems, MTA00B) and interleukin-1 beta (IL-1β) (R&D Systems, MLB00C) were measured with ELISA kits to determine inflammation.

### 2.6. Statistical Analysis

Results were expressed as mean ± standard error of mean (SEM). Significant results between groups were determined with independent two-tailed *t*-tests. Differences were considered significant if the *p*-value < 0.05.

## 3. Results

### 3.1. Body Weight and Food Intake

**Figure 2:**
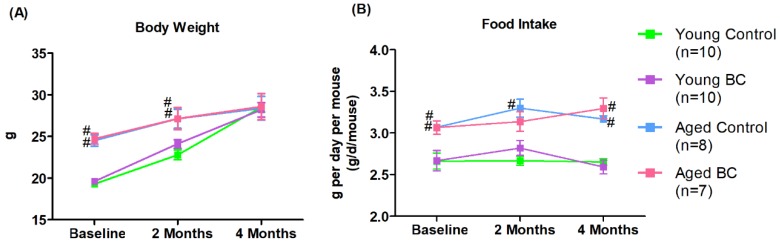
Comparison of mean body weight and daily food intake in young and aged mice consuming a control or blackcurrant (1% *w*/*w*) diet for 4 months. (**A**) Mean body weight change over four months. (**B**) Mean daily food intake per mouse. Values are reported as mean ± SEM. Data were analyzed using an independent *t*-test. Young control (*n* = 10), young BC (*n* = 10), aged control (*n* = 8), and aged BC (*n* = 7). # Indicates significant difference by age in mice consuming the same diet. BC, blackcurrant.

Young mice from both treatment groups weighed significantly less than their aged counterparts at baseline (week 0) and at 2 months but caught up in weight by 4 months (Figure 2A). Average daily food intake per mouse was significantly lower in young mice compared to their aged counterparts at all time points with the exception of the BC treatment group at 2 months (Figure 2B). There was no significant difference in body weight or average daily food intake between the control and BC treatments groups in either the young or aged mice.

### 3.2. Bone µCT Analysis

**Figure 3:**
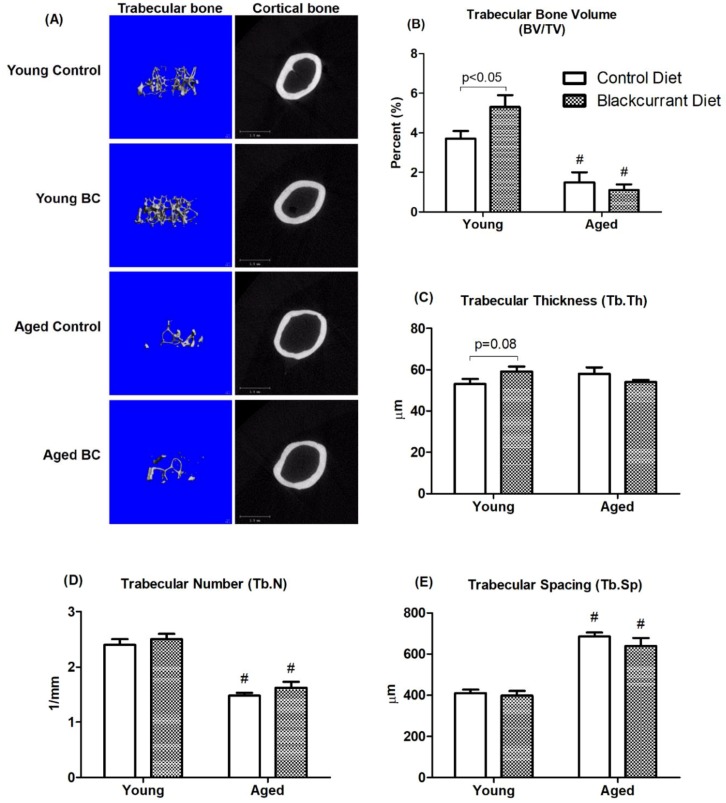
MicroCT images and analysis of femurs in young and aged mice consuming a control or blackcurrant (1% *w*/*w*) diet for 4 months. (**A**) Three-dimensional images of trabecular and cortical bone representative of each treatment group. (**B**–**E**) Trabecular bone parameters. Values are reported as mean ± SEM. Data were analyzed using an independent *t*-test. Young control (*n* = 10), young BC (*n* = 10), aged control (*n* = 8), and aged BC (*n* = 7). # Indicates significant difference by age in mice consuming the same diet. BC, blackcurrant.

The treatment effects of BC on trabecular and cortical bone were evaluated with µCT. Three-dimensional representative images of the right femur following four months of treatment are shown in Figure 3. As expected, aged mice had significantly lower trabecular BV/TV compared to young mice (Figure 3A,B). This reduction was consistent with decreased Tb.N and increased Tb.Sp (Figure 3D,E). In young mice, BC supplementation increased trabecular BV/TV by 43.2% compared to controls (*p* < 0.05) (Figure 3A,B). This effect was consistent with a trend towards increased Tb.Th with BC supplementation compared to controls (*p* = 0.08) in young mice (Figure 3C). In aged mice, BC supplementation did not appear to have an effect on trabecular bone parameters compared to controls. No significant changes were observed in cortical parameters in both young and aged mice.

### 3.3. Bone Histomorphometric Analysis

**Figure 4:**
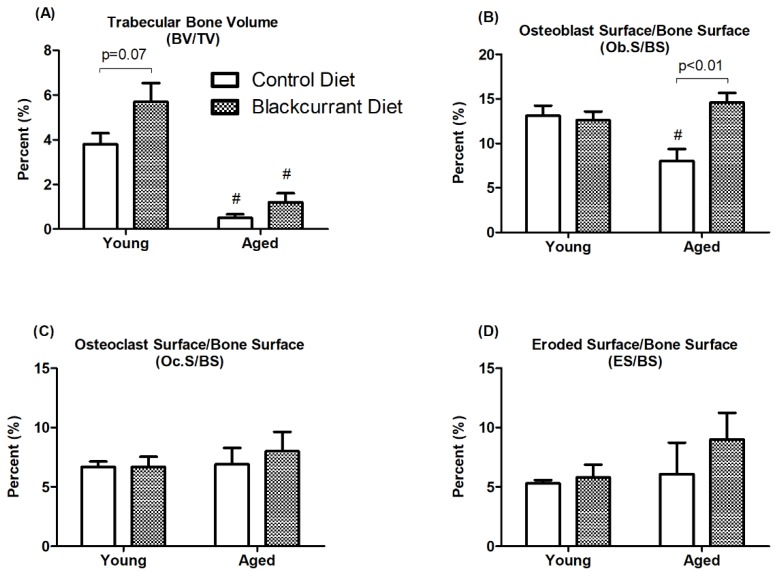
Histomorphometric analysis of femurs in young and aged mice consuming a control or blackcurrant (1% *w*/*w*) diet for 4 months. Values are reported as mean ± SEM. Data were analyzed using an independent *t*-test. Young control (*n* = 10), young BC (*n* = 10), aged control (*n* = 8), and aged BC (*n* = 7). # Indicates significant difference by age in mice consuming the same diet.

Static histomorphometric analysis was performed on the left femur to further evaluate the effect of BC supplementation on osteoblasts and osteoclasts. The aged mice from both treatment groups had significantly reduced trabecular BV/TV compared to the young mice (*p* < 0.05). BC consumption increased trabecular BV/TV in young mice compared to controls, though this effect was not statistically significant (*p* = 0.07) (Figure 4A). In aged mice, BC supplementation increased Ob.S/BS by 82.5% compared to controls (*p* < 0.01) (Figure 4B). BC consumption did not alter Ob.S/BS in young mice. BC supplementation appeared to have no effect on Oc.S/BS or ES/BS in either young or aged mice (Figure 4C,D).

### 3.4. DXA Analysis 

**Figure 5:**
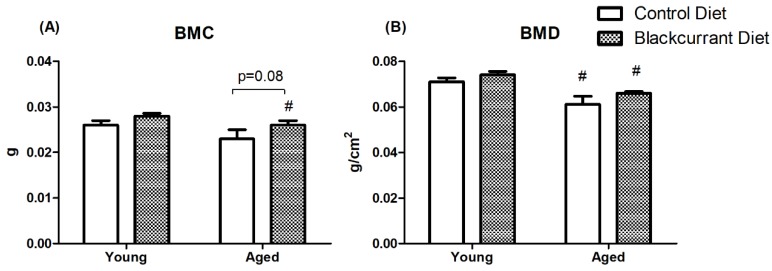
Dual-energy X-ray absorptiometry (DXA) analysis of (**A**) bone mineral content and (**B**) bone mineral density of femurs in young and aged mice consuming a control or blackcurrant (1% *w*/*w*) diet for 4 months. Values are reported as mean ± SEM. Data were analyzed using an independent *t*-test. Young control (*n* = 10), young BC (*n* = 10), aged control (*n* = 7), and aged BC (*n* = 7). # Indicates significant difference by age in mice consuming the same diet. BMC, bone mineral content; BMD, bone mineral density.

DXA analysis revealed that BMC and BMD were significantly reduced in aged mice indicating that aged mice had significantly reduced cortical bone compared to young mice. Blackcurrant consumption did not affect BMC and BMD in both young and aged mice (Figure 5).

### 3.5. Humerus Homogenate Biomarkers

**Figure 6:**
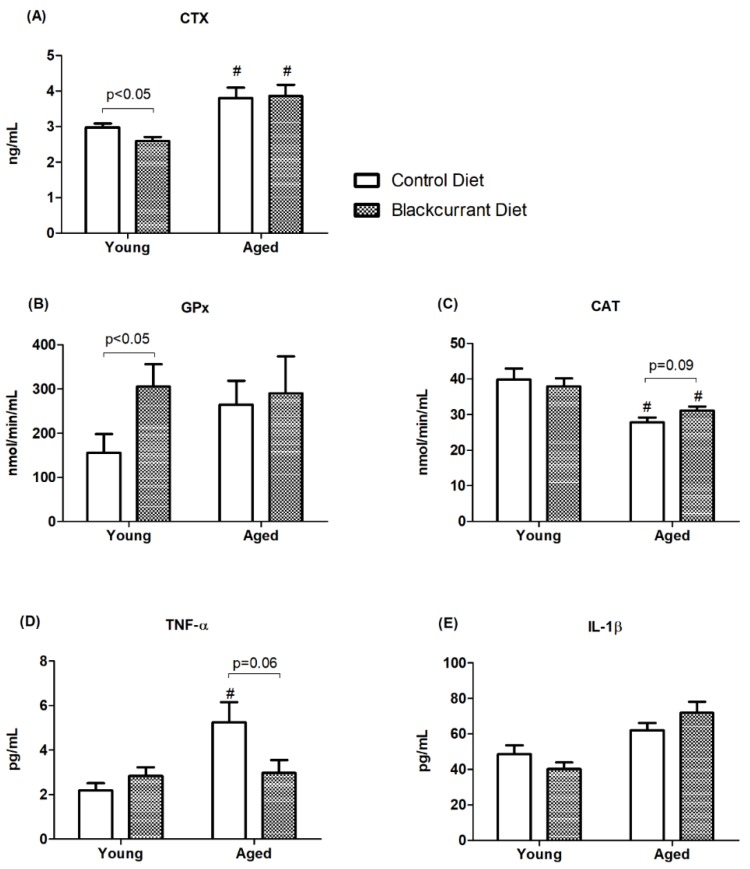
Bone homogenate biomarkers measured in young and aged mice consuming a control or blackcurrant (1% *w*/*w*) diet for 4 months. (**A**) Bone resorption marker CTX. (**B**,**C**) Antioxidant enzyme activity. (**D**,**E**) Pro-inflammatory cytokines. Values are reported as mean ± SEM. Data were analyzed using an independent *t*-test. Young control (*n* = 10), young BC (*n* = 10), aged control (*n* = 8), and aged BC (*n* = 7). # Indicates significant difference by age in mice consuming the same diet. CTX, C-terminal telopeptide of type I collagen; GPx, glutathione peroxidase; CAT, catalase; TNF-α, tumor necrosis factor α; IL-1β, interleukin-1 beta.

To further assess bone turnover, the bone resorption marker CTX was measured in the humerus homogenate. BC consumption decreased CTX by 12.4% compared to controls in young mice (*p* < 0.05) (Figure 6A), which is consistent with the increased trabecular BV/TV seen in the µCT and histomorphometric analyses. Notably, BC supplementation had no effect on CTX in aged mice.

The activities of the antioxidant defense enzymes GPx and CAT were measured to assess the relation between antioxidant capacity and bone turnover. In young mice, BC consumption increased GPx activity by 96.4% (*p* < 0.05) (Figure 6B), whereas in aged mice there was no effect. In aged mice consuming BC, CAT activity increased by 12% compared to controls, but this effect was not statistically significant (*p* = 0.09) (Figure 6C).

The pro-inflammatory cytokines TNF-α and IL-1β were measured in humerus homogenate to evaluate the relationship between inflammation and bone resorption. Aged control mice had significantly higher TNF-α concentrations than young controls (aged control, 5.3 ± 0.9 pg/mL vs. young control, 2.2 ± 0.3 pg/mL) (*p* < 0.01), indicating increased inflammation due to aging. The aged mice consuming BC had a 43.3% lower TNF-α concentration compared to controls (*p* = 0.06) (Figure 6D), suggesting an anti-inflammatory effect of BC consumption. BC supplementation appeared to have no effect on IL-1β concentrations in either the young or aged mice.

## 4. Discussion

Age-related bone loss results from an imbalance of bone remodeling, where bone resorption outweighs bone formation [27]. It begins as early as one’s 30s [28] and accelerates with sex steroid deficiency, such as in the case of menopause [29], oxidative stress [9,30,31], and inflammation [8]. In this study we sought to determine if BC consumption can attenuate aging-related bone loss in mice and to explore the underlying mechanisms.

In the µCT analysis of femoral trabecular bone, aged mice had weaker bone microarchitecture compared to young mice. BC consumption significantly increased bone mass in young mice. Additionally, this coincided with a reduction in the bone resorption marker CTX, indicating that the increased trabecular bone mass may be related to attenuated bone resorption. Interestingly, BC consumption appeared to have no effect on trabecular BV/TV in aged mice. We initially hypothesized that BC supplementation would improve bone mass in both young and aged mice, but we found that bone mass did not improve in aged mice. The lack of a response in the 18-month-old mice used in this study may be related to their advanced age and relatively high degree of bone loss at baseline. Female mice begin to lose trabecular bone at 2 months old, with 94% bone loss by 14 months old [32]. This means that the trabecular bone of the aged mice may have been too weak to rescue even prior to the initiation of dietary intervention with BC. Halloran et al. [23] observed similar results, where six months of dried plum supplementation (control diet plus 15% dried plum by weight) significantly improved the trabecular BV/TV of 6-month-old male mice but not 18-month-old mice. However, consumption of a higher concentration of dried plum (25%) improved trabecular BV/TV in both the 6-month-old and 18-month-old mice, indicating that bones weakened due to advanced aging may respond to a sufficient amount of dietary polyphenols. Furthermore, the increase in Ob.S/BS in aged mice consuming BC indicates that although osteoblast function was improved [33], it was perhaps insufficient to reverse advanced aging-associated bone loss.

The accumulation of oxidative stress from the aging process is known to contribute to bone loss. As age increases, sex hormone production decreases, resulting in diminished oxidative defense systems [34]. This weakening of cells’ oxygen-scavenging capabilities increases oxidative stress produced from reactive oxygen species (ROS), which in turn stimulates osteoclast differentiation [35], induces osteoblast and osteocyte apoptosis [36], and inhibits osteoblastogenesis [10], all resulting in suppressed bone formation. Bone formation rate also decreases due to the oxidation of lipids generating ligands that activate peroxisome proliferator-activated receptor gamma, attenuating Wnt signaling [10]. Hydrogen peroxide, an ROS, has been shown to directly induce bone resorption by stimulating osteoclast formation and function [37]. Thus, due to the implication of oxidative stress in aging-related bone loss, improving antioxidant defense is a logical goal.

GPx is the predominant antioxidant enzyme expressed by osteoclasts and is responsible for degrading hydrogen peroxide [20]. Lean et al. [20] observed that osteoclast precursor RAW 264.7 macrophages overexpressing GPx did not differentiate into osteoclasts when induced with receptor activator of nuclear factor κB ligand. Additionally, they found that OVX mice that were administered pegylated CAT for two weeks had a higher trabecular BV/TV compared to OVX mice in the control group, demonstrating the bone-protective effect of antioxidant enzymes. In this study, BC consumption increased GPx activity in young mice and decreased bone resorption as measured by CTX concentrations. As this was met with increased trabecular bone mass, it is possible that the improved antioxidant defense inhibited bone resorption and ultimately improved bone mass. CAT activity decreased with aging in both treatment groups and only moderately increased with BC consumption in aged mice. As antioxidant defense enzymes, GPx and CAT were both expected to follow similar trends; it was thus surprising that GPx activity increased in young mice while CAT activity did not. It appears then that GPx activity may respond more favorably to BC consumption, at least in the confines of this study. Collectively, this substantiates the notion that oxidative stress promotes bone resorption activity and that improving antioxidant defense may protect from its deleterious effects on bone health.

Aging increases chronic low-grade inflammation due to several factors, such as increased generation of ROS, cell senescence, decreased gut microbial diversity and weakening of the oral and gut mucosa, and dysregulation of the immune system [38], which contributes to bone loss. In the current study, the concentration of the pro-inflammatory cytokine TNF-α increased with aging in mice consuming the control diet. Excessive inflammation is associated with bone loss due to increased osteoclast-mediated bone resorption [8,38,39]. Through the modulation of osteoclast formation and activity, pro-inflammatory cytokines induce bone resorption: TNF-α and IL-1β increase osteoclast formation [40] and activity [41], and inhibit osteoclast apoptosis, prolonging osteoclast lifespan [40]. Taken together, elevated concentrations of pro-inflammatory cytokines may promote bone loss in conditions such as postmenopausal osteoporosis and inflammatory osteolysis. Furthermore, hydrogen peroxide exposure increases the expression of pro-inflammatory cytokines [20], implicating oxidative stress and inflammation as promoters of bone resorption. In this study, BC consumption decreased TNF-α concentrations in aged mice but not in young mice, though this effect was only moderately significant. This possibly suggests that the anti-inflammatory effect of BC may be more potent in the context of a pro-inflammatory state. Shahnazari et al. [17] observed a reduction in TNF-α and IL-1β concentrations and increased trabecular bone mass when mice were fed anthocyanin-rich dried plum (25% *w*/*w*), while IL-1β concentrations were unaffected by BC in our study. This disparity in results may be attributed to the difference in concentrations of the fruits used in the diet, which is important to consider when testing and evaluating the efficacy of dietary treatments. It is possible that the concentration of flavonoids in the BC used for this study was not sufficient to reduce IL-1β concentrations. Additionally, since aging did not significantly increase IL-1β concentrations, any observed anti-inflammatory effect of BC would be blunted. Anthocyanins have been found to inhibit mRNA transcription of IL-1β by inhibiting nuclear factor κB (NF-κB) [42] and TNF-α [22], as well as suppressing NF-κB activity [16,42] in vitro; and as such, a higher concentration of anthocyanins would be expected to have a more pronounced anti-inflammatory effect.

A strength of this study is the use of an aging mouse model to induce age-related bone loss as opposed to using an OVX model. This fully mimics the effects of aging and thus has wider applicability than just estrogen deficiency that is provided by OVX. Another strength is that evaluating changes in bone mass using anthocyanin-rich BC extract in the diet, compared to using isolated compounds, allows for better translatability for food-based studies. However, this also means that it is more difficult to determine the exact component(s) responsible for the observed effects. Additionally, µCT, histomorphometric, and DXA analyses were performed only on the femur. Including additional bones, such as vertebrae, in future studies would expand the width of bone considered and potentially strengthen results.

In conclusion, the results of this study suggest that BC consumption improves trabecular bone mass in young mice that have not already lost a substantial amount of bone mass due to aging. The underlying mechanism appears to be related to improved antioxidant defense, as evidenced by the increase in GPx activity, though the exact mechanism remains unclear. BC was able to moderately reduce inflammation in aged mice, but was unable to affect bone mass, presumably because there was very little bone to rescue. We have determined that early consumption of BC is crucial in preventing age-related bone loss and that the beneficial effects on bone morphology may not be apparent if the dietary intervention is initiated late in age. In the search for non-pharmacological treatments for age-related bone loss, this both highlights anthocyanin-rich food as potential bone-protective dietary agents as well as emphasizes the importance of early intervention. Future studies are needed to determine the optimal treatment timing and dosage at which BC and other anthocyanin-rich foods may be effective for attenuating age-related bone loss.

## References

[1] Demontiero O., Vidal C., Duque G. (2012). Aging and bone loss: New insights for the clinician. Ther. Adv. Musculoskelet. Dis..

[2] Vedi S., Compston J., Webb A., Tighe J. (1984). Histomorphometric analysis of dynamic parameters of trabecular bone formation in the iliac crest of normal British subjects. Metab. Bone Dis. Relat. Res..

[3] Ericksen E. (1986). Normal and pathological remodeling of human trabecular bone: Three dimensional reconstruction of the remodelling sequence in normals and in metabolic disease. Endoc. Rev..

[4] Lips P., Courpron P., Meunier P. (1978). Mean wall thickness of trabecular bone packets in the human iliac crest: Changes with age. Calcif. Tissue Res..

[5] Bjørnerem Å., Ghasem-Zadeh A., Bui M., Wang X., Rantzau C., Nguyen T., Hopper J., Zebaze R., Seeman E. (2011). Remodeling markers are associated with larger intracortical surface area but smaller trabecular surface area: A twin study. Bone.

[6] Mosekilde L., Mosekilde L. (1990). Sex differences in age-related changes in vertebral body size, density and biomechanical competence in normal individuals. Bone.

[7] Zebaze R., Ghasem-Zadeh A., Bohte A., Iuliano-Burns S., Mirams M., Price R., Mackie E., Seeman E. (2010). Intracortical remodelling and porosity in the distal radius and post-mortem femurs of women: A cross-sectional study. Lancet.

[8] Lacativa P.G.S., De Farias M.L.F. (2010). Osteoporosis and inflammation. Arq. Bras. Endocrinol. Metab..

[9] Shen C., Chyu M., Wang J. (2013). Tea and bone health: Steps forward in translational nutrition. Am. J. Clin. Nutr..

[10] Manolagas S.C. (2010). From estrogen-centric to aging and oxidative stress: A revised perspective of the pathogenesis of osteoporosis. Endocr. Rev..

[11] Qaseem A., Forciea M.A., McLean R.M., Denberg T.D. (2017). Treatment of low bone density or osteoporosis to prevent fractures in men and women: A clinical practice guideline update from the American college of physicians. Ann. Intern. Med..

[12] Rossini M., Adami S., Bertoldo F., Diacinti D., Gatti D., Giannini S., Giusti A., Malavolta N., Minisola S., Osella G. (2016). Guidelines for the diagnosis, prevention and management of osteoporosis. Reumatismo.

[13] Kanis J.A., McCloskey E.V., Johansson H., Cooper C., Rizzoli R., Reginster J.Y. (2013). European guidance for the diagnosis and management of osteoporosis in postmenopausal women. Osteoporos Int..

[14] Akkawi I., Zmerly H. (2018). Osteoporosis: Current Concepts. Joints.

[15] Lee S.G., Vance T.M., Nam T.G., Kim D.O., Koo S.I., Chun O.K. (2015). Contribution of Anthocyanin Composition to Total Antioxidant Capacity of Berries. Plant Foods Hum. Nutr..

[16] Moriwaki S., Suzuki K., Muramatsu M., Nomura A., Inoue F., Into T., Yoshiko Y., Niida S. (2014). Delphinidin, one of the major anthocyanidins, prevents bone loss through the inhibition of excessive osteoclastogenesis in osteoporosis model mice. PLoS ONE.

[17] Shahnazari M., Turner R.T., Iwaniec U.T., Wronski T.J., Li M., Ferruzzi M.G., Nissenson R.A., Halloran B.P. (2016). Dietary dried plum increases bone mass, suppresses proinflammatory cytokines and promotes attainment of peak bone mass in male mice. J. Nutr. Biochem. Elsevier Inc..

[18] Zheng X., Mun S., Lee S.G., Vance T.M., Hubert P., Koo S.I., Lee S.-K., Chun O.K. (2016). Anthocyanin-Rich Blackcurrant Extract Attenuates Ovariectomy-Induced Bone Loss in Mice. J. Med. Food.

[19] Kaume L., Gilbert W., Smith B.J., Devareddy L. (2015). Cyanidin 3-O-β-d-Glucoside Improves Bone Indices. J. Med. Food.

[20] Lean J.M., Jagger C.J., Kirstein B., Fuller K., Chambers T.J. (2005). Hydrogen peroxide is essential for estrogen-deficiency bone loss and osteoclast formation. Endocrinology.

[21] Lee S.G., Kim B., Soung D.Y., Vance T., Lee J.S., Lee J., Koo S.I., Kim D.-O., Drissi H., Chun O.K. (2015). Relationship Between Oxidative Stress and Bone Mass in Obesity and Effects of Berry Supplementation on Bone Remodeling in Obese Male Mice: An Exploratory Study. J. Med. Food.

[22] Benn T., Kim B., Park Y.K., Wegner C.J., Harness E., Nam T.G., Kim D.O., Lee J.S., Lee J.Y. (2014). Polyphenol-rich blackcurrant extract prevents inflammation in diet-induced obese mice. J. Nutr. Biochem..

[23] Halloran B.P., Wronski T.J., VonHerzen D.C., Chu V., Xia X., Pingel J.E., Williams A.A., Smith B.J. (2010). Dietary Dried Plum Increases Bone Mass in Adult and Aged Male Mice. J. Nutr..

[24] Smith B.J., Graef J.L., Wronski T.J., Rendina E., Williams A.A., Clark K.A., Clarke S.L., Lucas E.A., Halloran B.P. (2014). Effects of dried plum supplementation on bone metabolism in adult C57BL/6 male mice. Calcif. Tissue Int..

[25] Bouxsein M.L., Boyd S.K., Christiansen B.A., Guldberg R.E., Jepsen K.J., Müller R. (2010). Guidelines for assessment of bone microstructure in rodents using micro-computed tomography. J. Bone Miner Res..

[26] Parfitt A., Drezner M., Glorieux F., Kanis J., Malluche H., Meunier P., Ott S., Recker R. (1987). Bone histomorphometry: Standardization of nomenclature, symbols, and units. Report of the ASBMR Histomorphometry Nomenclature Committee. J. Bone Miner Res..

[27] Plotkin L.I., Laird D.W., Amedee J. (2016). Role of connexins and pannexins during ontogeny, regeneration, and pathologies of bone. BMC Cell Biol..

[28] Looker A.C., Wahner H.W., Dunn W.L., Calvo M.S., Harris T.B., Heyse S.P., Johnston C.C., Lindsay R. (1998). Updated data on proximal femur bone mineral levels of US adults. Osteoporos Int..

[29] Khosla S., Riggs B.L. (2005). Pathophysiology of age-related bone loss and osteoporosis. Endocrinol. Metab. Clin. N. Am..

[30] Syed F.A., Ng A.C. (2010). The pathophysiology of the aging skeleton. Curr. Osteoporos Rep..

[31] Baek K.H., Oh K.W., Lee W.Y., Lee S.S., Kim M.K., Kwon H.S., Rhee E.J., Han J.H., Song K.H., Cha B.Y. (2010). Association of oxidative stress with postmenopausal osteoporosis and the effects of hydrogen peroxide on osteoclast formation in human bone marrow cell cultures. Calcif.Tissue Int..

[32] Glatt V., Canalis E., Stadmeyer L., Bouxsein M.L. (2007). Age-related changes in trabecular architecture differ in female and male C57BL/6J mice. J. Bone Miner Res..

[33] Byers R., Denton J., Hoyland J., Freemont A. (1997). Differential patterns of osteoblast dysfunction in trabecular bone in patients with established osteoporosis. J. Clin. Pathol..

[34] Russell S.J., Kahn C.R. (2007). Endocrine regulation of ageing. Nat. Rev. Mol. Cell Biol..

[35] Shimizu S., Matsushita H., Morii Y., Ohyama Y., Morita N., Tachibana R., Watanabe K., Wakatsuki A. (2018). Effect of anthocyanin-rich bilberry extract on bone metabolism in ovariectomized rats. Biomed. Rep..

[36] Bai X.C., Lu D., Bai J., Zheng H., Ke Z.Y., Li X.M., Luo S.Q. (2004). Oxidative stress inhibits osteoblastic differentiation of bone cells by ERK and NF-κB. Biochem. Biophys. Res. Commun..

[37] Bax B.E., Alam A.S.M.T., Banerji B., Bax C.M.R., Bevis P.J.R., Stevens C.R., Moonga B.S., Blake D.R., Zaidi M. (1992). Stimulation of osteoclastic bone resorption by hydrogen peroxide. Biochem. Biophys. Res. Commun..

[38] Sanada F., Taniyama Y., Muratsu J., Otsu R., Shimizu H., Rakugi H., Morishita R. (2018). Source of Chronic Inflammation in Aging. Front. Cardiovasc. Med..

[39] Mundy G. (2007). Osteoporosis and inflammation. Nutr. Rev..

[40] Weitzmann M.N., Pacifici R. (2005). The role of T lymphocytes in bone metabolism. Immunol. Rev..

[41] Teitelbaum S.L. (2007). Osteoclasts: What do they do and how do they do it?. Am. J. Pathol..

[42] Lee S.G., Kim B., Yang Y., Pham T.X., Park Y.K., Manatou J., Koo S.I., Chun O.K., Lee J.Y. (2014). Berry anthocyanins suppress the expression and secretion of proinflammatory mediators in macrophages by inhibiting nuclear translocation of NF-κB independent of NRF2-mediated mechanism. J. Nutr. Biochem..

